# Nasal Alar Necrosis Following Hyaluronic Acid Injection into Nasolabial Folds: A Case Report

**Published:** 2015-01

**Authors:** Ali Manafi, Behrooz Barikbin, Amir Manafi, Zahra Sadat Hamedi, Shokoofeh Ahmadi Moghadam

**Affiliations:** 1 Department of Plastic Surgery, Iran University of Medical Sciences, Tehran, Iran; 2 Shohada Tajrish Hospital, Laser Application in Medical Sciences Research Center, Tehran, Iran; 3 Department of Medicine, Shahid Beheshti University of Medical Sciences, Tehran, Iran;; 4 School of Medicine, Islamic Azad University, Tehran Branch, Tehran, Iran

**Keywords:** Hyaluronic acid, Soft tissue, Injection, Alar necrosis

## Abstract

Injection of synthetic fillers for soft tissue augmentation is increasing over the last decade. One of the most common materials used is hyaluronic acid (HA) that is safe and temporary filler for soft tissue augmentation. We present a case of 54-year-old female who experienced vascular occlusion and nasal alar necrosis following HA injection to the nasolabial folds. She suffered from pain, necrosis, infection, and alar loss that finally required a reconstructive surgery for cosmetic appearance of the nose. The case highlights the importance of proper injection technique by an anesthesiologist, as well as the need for immediate recognition and treatment of vascular occlusion.

## INTRODUCTION

Soft tissue augmentation with temporary dermal fillers is one of the most common cosmetic procedures.^1^ There are a lot of materials that have been used as a dermal fillers for this purpose previously,^2^ but hyaluronic acid (HA) fillers have become the material of choice for temporary augmentation. HA fillers have some advantages, such as longer lasting and less immunogenic reactions, and finally can be hydrolysed by hyaluronidase enzyme.^3^

Some complications associated with its use have been reported, and most of them are rare and benign.^4^ Other complications were reported as follows: infection, nodules, hypersensitivity reactions, and arterial compromise.^5^ The most severe and early-occurring complication is tissue necrosis due to embolization of specific vessel and obstruction of the vessel by the filler material which can cause ischemia and tissue necrosis.^1^ There are few reports of nasal alar area necrosis after HA injection in nasolabial folds. Here, we describe a case of alar necrosis after HA injection.

## CASE REPORT

**Fig. 1:**
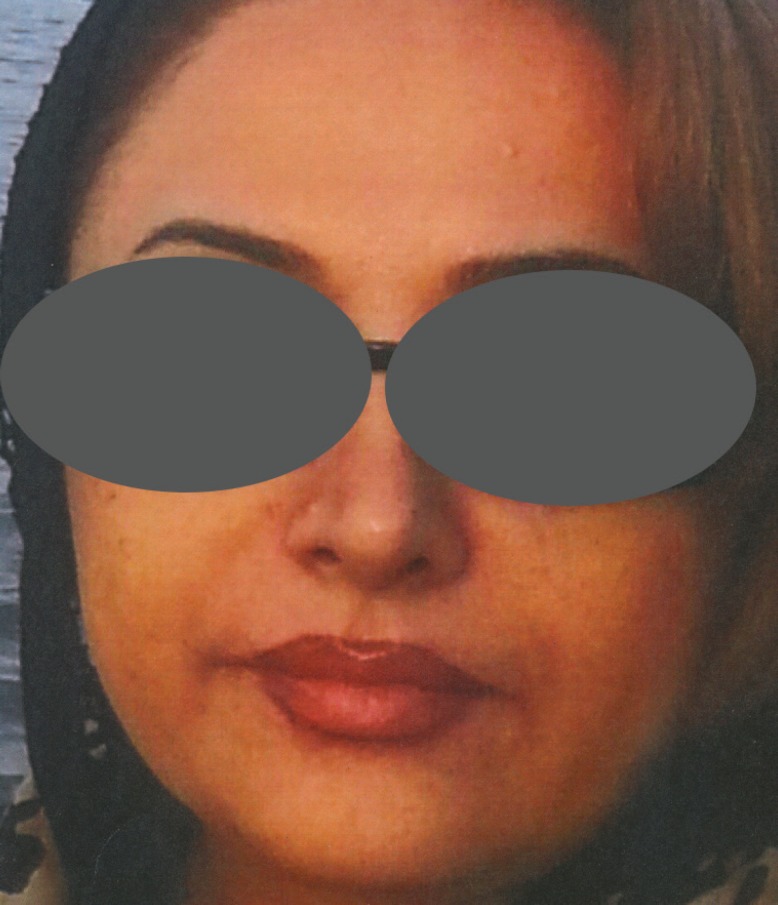
An amatory photo before HA injection shows normal nasal alar structures

**Fig. 2:**
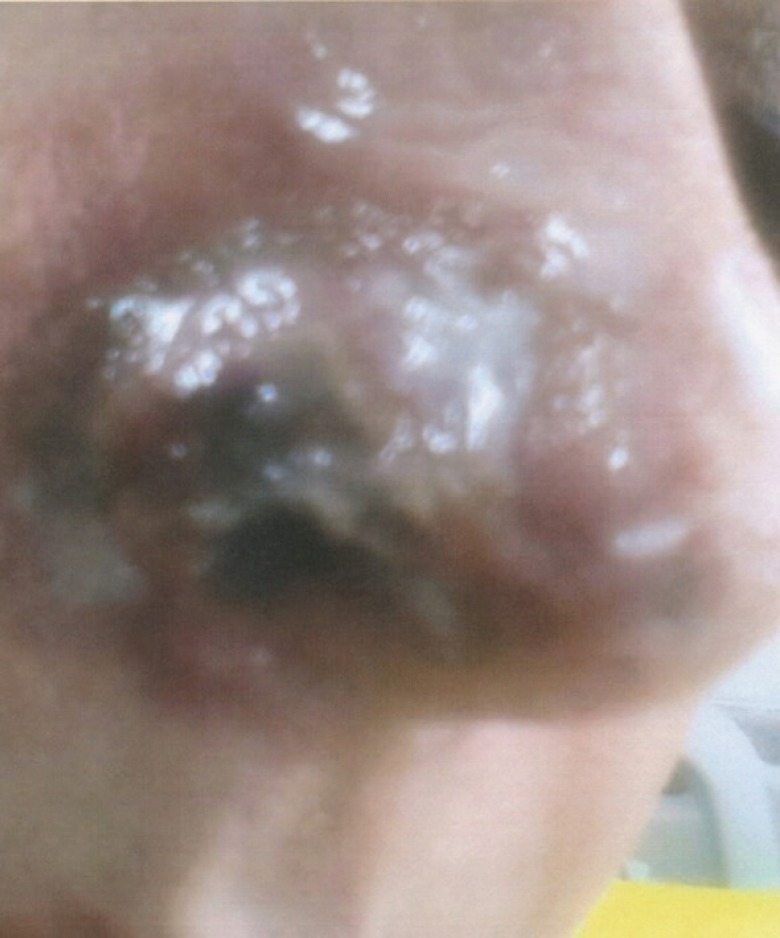
Right nasal alar partial necrosis, 3 weeks after HA injection to right nasolabial fold

**Fig. 3:**
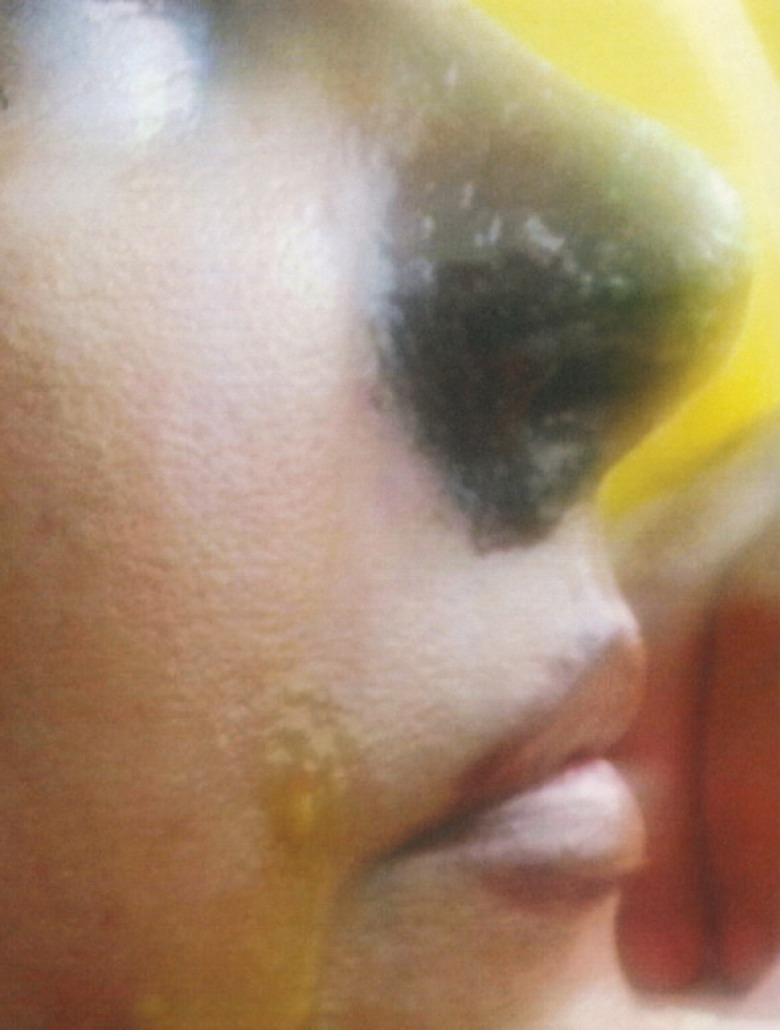
Forty days after HA injection, the necrotic areas demarcated in alar region

**Fig. 4:**
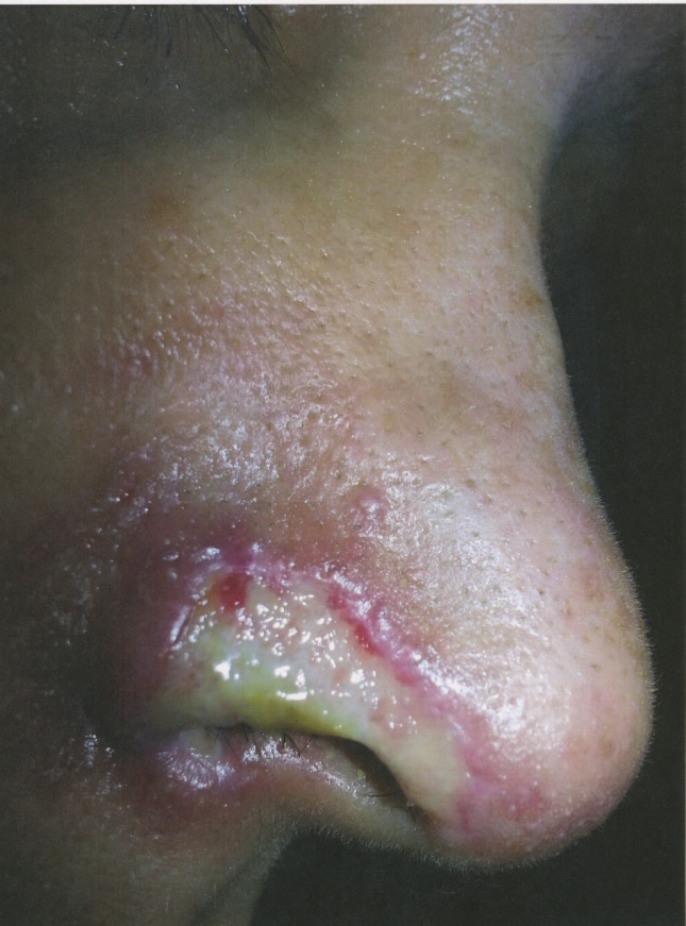
Two months after injury, the gross necrosis tissue sloughed and its bed inflamed and was fibrinous

A 54-years-old Iranian woman underwent injection of HA fillers into nasolabial folds for wrinkle correction of this region by an anaesthesiologist (Figure 1). A few minutes after injection of HA, she noticed pain and reddish discoloration in the right side of the nose. By the third day from the onset, blisters appeared at the right nasal ala (Figure 2). Then a black area in the right nasal ala appeared which represent skin necrosis in this area, the necrotic areas well demarcated (Figure 3). With passing time and wound care the grossly necrosed part sloughed and the bed was tidy and inflamed (Figure 4). She had developed localized and then widespread mid facial swelling and infection, resulted into hospitalization of her due to a cavernous sinous thrombosis. After 5 days, she was discharged with improvement of her signs and symptoms.

**Fig. 5:**
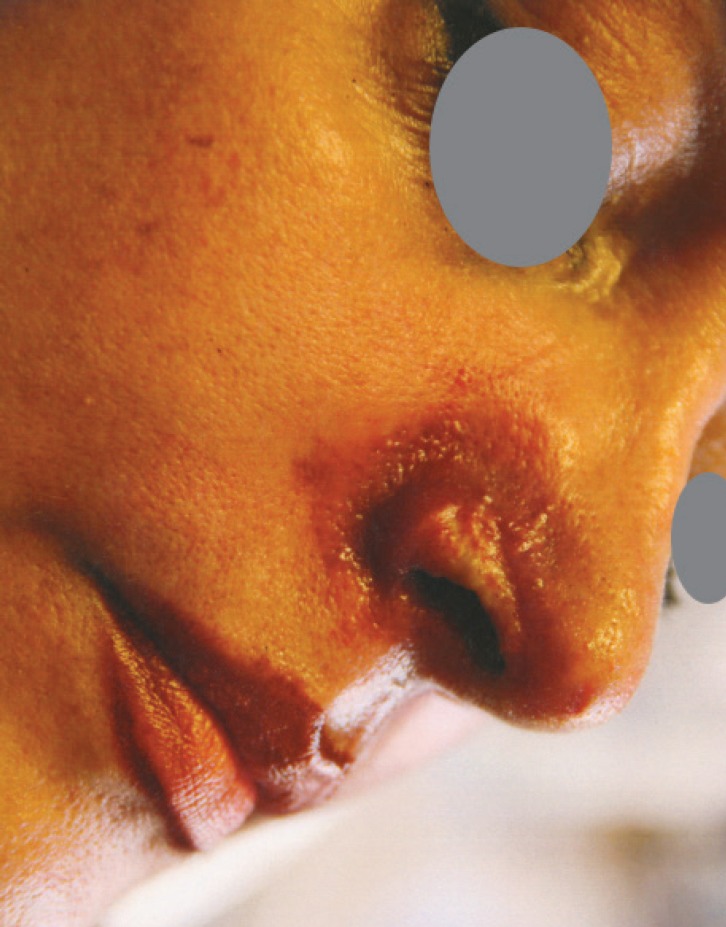
Three months after injury, when she was visited by plastic surgeon, the bed of right nasal alar region and perinasal area were inflamed and fragile

**Fig. 6:**
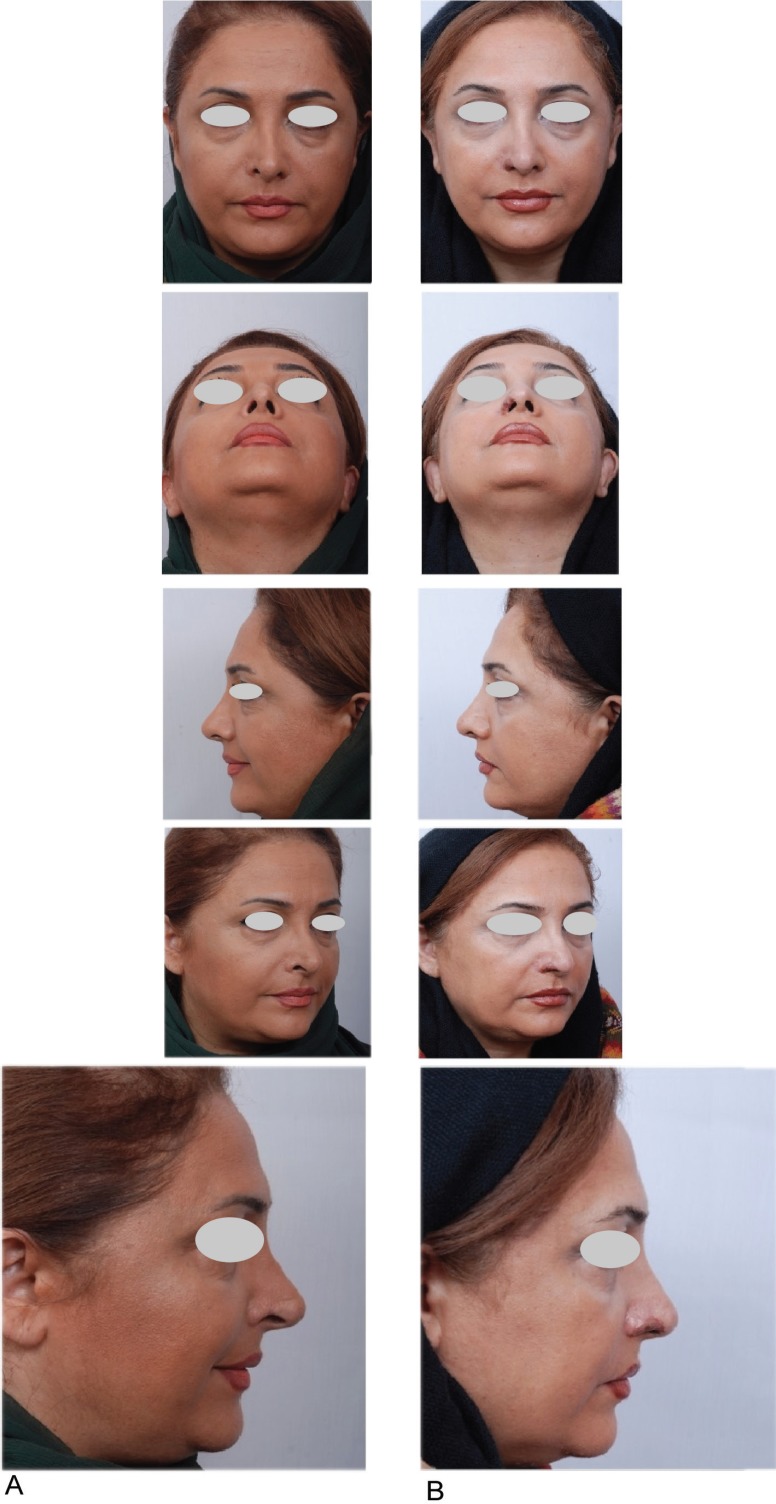
A (Left column): One year after injury and before reconstruction, B: (Right column) Three weeks after the reconstruction with composite graft from the right auricular helical root which showed excellent take

When our plastic surgeon visited the patient the right nasal alar area has had skin tissue shortage and it was inflamed. She was advised to tolerate it up to improvement of the inflammation and wound maturation (Figure 5). After one year and subsidence of skin inflammation and irritation, a composite graft taken from the helical root of her right auricule was grafted to the residual skin defect (Figure 6).

## DISCUSSION

HA was first named by Larl Meyer, who first found this polysaccharide in the ‘vitreous humor’ of the bovine eye. It was subsequently found in all species, including man. HA is in fact a naturally found glycosaminoglycan constructing the extracellular matrix of connective tissues.^6^ There are several complications with HA filler injection including inflammation, erythema and nodule formation.^7^ The most serious and feared complications are vascular embolization and skin necrosis which is likely caused by interruption of the vascular supply to the area due to direct injury to the vasculature and arterial obstruction by large molecular HA filler secondary to its hydrophilic action.^3^^,^^8^

An excessive amount of dermal fillers can cause vascular compression resulting into a reduction in skin perfusion.^9^ Delayed injection necrosis through vascular compression from hyaluronic acid has been described previously.^10^ Minimizing this risk of aspiration prior to injection is recommended. Also it has been suggested that use of small amounts of filler and a suitable injection technique may reduce the risk of the complications.^3^ Early diagnosis of vascular compromise and even vascular necrosis after filler injection may improve the outcome of wound healing.^11^

The two danger zones which are vulnerable to tissue necrosis are the glabella and nasal ala.^2^^,^^12^ Similar to the glabellar region, the nasal ala is a particular region strictly dependent on a single arterial branch. Although accidental intra-arterial injection of dermal fillers has been a rare condition, the potential risk of vascular embolization should be considered in case of injection into subcutis of the glabellar region, the nasal ala and nasolabial folds.^3^ Therefore, compression of the facial artery from nasolabial folds injection or compression of the angular artery, at the alar rim could elucidate nasal tip or alar necrosis.^13^

Manafi et al. (2012) reported successful use of platelet-rich plasma on cartilage grafts in rabbits as an animal model.^14^ In 2013, Manafi et al. denoted good results for application of auricular composite graft in rhinoplasty armamentarium.^15^ In Rabbit, a comparasion was undertaken for graft resorption between three techniques of diced cartilage using surgical blade, electrical grinder and grater showing no statistically difference between groups and the use of both techniques in reconstructive and in aesthetic cases was recommended.^16^

In this case, despite the avoidance of direct filler injection into nasal ala, the right alar skin and cartilage became necrotic. In our case report, an anesthesiologist performed the injection procedure and the patient visited our dermatologist one month later due to the infection, necrosis and cosmetic complications. In the examination of the right nasal alar region, a permanent disfiguring asymmetry of the affected tissue was noticed while any initial management with hyaluronidase could not be performed. Wound care was continued and plastic surgical consultation was undertaken 6 months after injection. At her visit, the risks and benefits of all methods of reconstructions were explained to her, and she was recommended to wait for wound maturation prior to reconstruction. After one year, reconstruction surgery was undertaken. 

In summary, we report a case of right nasal alar necrosis due to HA injection into nasolabial folds that resulted into scar and assymetric figure of the nose. She was successfully managed with composite graft of the auricular area. 

## CONFLICT OF INTEREST

The authors declare no conflict of interest.

## Note

Please cite this paper as:

Manafi A, Barikbin B, Manafi A, Hamedi ZS, Ahmadi Moghadam S. Nasal Alar Necrosis Following Hyaluronic Acid Injection into Nasolabial Folds: A Case Report. World J Plast Surg 2015;4(1):74-78.
